# Smartwatch-Derived Digital Phenotypes Relate to Psychopathology Dimensions in Patients With Psychotic Spectrum Disorders: Longitudinal Observational Study

**DOI:** 10.2196/75774

**Published:** 2025-12-12

**Authors:** Vasiliki Garyfalli, Emmanouil Kalisperakis, Alexandros Smyrnis, Marina Lazaridi, Thomas Karantinos, Asimakis Mantas, Panagiotis P Filntisis, Niki Efthymiou, Athanasia Zlatintsi, Petros Maragos, Nikolaos Smyrnis

**Affiliations:** 1 Laboratory of Cognitive Neuroscience and Sensorimotor Control, University Mental Health Research Institute, Athens, Greece; 2 1st Department of Psychiatry, Eginitio Hospital, National and Kapodistrian University of Athens, Athens, Greece; 3 2nd Department of Psychiatry, University General Hospital "ATTIKON," National and Kapodistrian University of Athens, 1 Rimini St., Athens, 12462, Greece, 30 2105832426; 4 School of Electrical and Computer Engineering, National Technical University of Athens, Athens, Greece

**Keywords:** digital phenotype, sympathetic heart activation, parasympathetic heart activation, smartwatch, psychopathology dimension, schizophrenia, bipolar disorder

## Abstract

**Background:**

Digital phenotyping refers to the objective measurement of human behavior via devices such as smartphones or watches and constitutes a promising advancement in personalized medicine. Digital phenotypes derived from heart rate, mobility, or sleep schedule data have been used in psychiatry to either diagnose individuals with psychotic disorders or to predict relapse as a binary outcome. Machine learning models so far have achieved predictive accuracies that are significant but not large enough for clinical applications. This could hinge on broad clinical definitions, which encompass heterogeneous symptom and sign ensembles, thus hindering accurate classification. The 5-factor model for the Positive and Negative Syndrome Scale (PANSS), which entails 5 independently varying dimensions, is thought to better capture symptom variability. Using the specific definitions of this refined clinical taxonomy in combination with digital phenotypes could yield more precise results.

**Objective:**

This study aims to investigate potential links between digital phenotypes and each dimension of the 5-factor PANSS model. We also assess whether clinical, demographic, and medication variables confound said reactions.

**Methods:**

In the e-Prevention study, heart rate, accelerometer, gyroscope, and sleep schedule data were continuously collected via smartwatch for a maximum of 26 months in 38 patients with psychotic spectrum disorders. Obtaining the mean and SD for each patient-month resulted in a database consisting of more than 740 monthly data points. A linear mixed model analysis was used to ascertain connections between monthly aggregated heart rate and mobility features and the 5 symptom dimension scores of PANSS, obtained during monthly clinical interviews.

**Results:**

An increase in positive symptoms was associated with a decrease in heart interpulse variation during sleep (*t*_570.7_=−3.3, *P*<.001, *f*^2^=0.021), while an increase in negative symptoms was associated with a decrease in accelerometer (mean: *t*_22.1_=−3.1, *P*=.005, *f*^2^=0.042; SD: *t*_20_=−2.4, *P*=.03, *f*^2^=0.019), gyroscope (mean: *t*_22.9_=−2.8, *P*=.01, *f*^2^=0.016), and locomotive motor activity (*t*_17.2_=−2.4, *P*=.03, *f*^2^=0.016) during wakefulness. An increase in accelerometer (mean: *t*_564.4_=2.8, *P*=.005, *f*^2^=0.017; SD: *t*_551.6_=2.5, *P*=.01, *f*^2^=0.015) and gyroscope (mean: *t*_564.5_=3.2, *P*=.001, *f*^2^=0.022; SD: *t*_569.2_=2.8, *P*=.005, *f*^2^=0.017) motor activity during sleep was related to an increase in depression/anxiety symptoms as well as excitement/hostility symptoms (accelerometer SD: *t*_469.7_=3.2, *P*=.002, *f*^2^=0.031; gyroscope mean: *t*_497_=2.3, *P*=.03, *f*^2^=0.013; SD: *t*_507.7_=3.2, *P*=.001, *f*^2^=0.029). Excitement/hostility symptoms were further associated with an increase in normalized heart rate during sleep (*t*_368.2_=3.2, *P*=.001, *f*^2^=0.044) and reduced sleep:wake ratio (*t*_562_=−2.7, *P*=.007, *f*^2^=0.013). An increase in cognitive/disorganization symptoms was related to a decrease in the SD of normalized heart rate during wakefulness (*t*_574.5_=−3.5, *P*<.001, *f*^2^=0.013).

**Conclusions:**

This study provides evidence that biological changes assessed by continuous measurement of digital phenotypes could be characteristic of specific symptom clusters rather than entire diagnostic categories of psychotic disorders. These results support the use of digital phenotypes not only as a means for remote patient monitoring but also as concrete targets for biomarker research in psychotic disorders.

## Introduction

Psychotic spectrum disorders manifest in a variety of psychopathological symptoms and signs that vary across multiple time scales, ranging from hours or days (eg, the development of positive symptoms in an acute psychotic or manic episode) to months or even years (eg, the subtle increase of negative symptoms in remitted patients). In clinical practice, heterogeneous symptoms and signs are grouped in clusters to define categorical diagnoses of psychotic disorders (schizophrenia or bipolar disorder) that are used as invariant labels chronically attached to patients, guiding clinical decisions. The shortcomings of such an approach in capturing the dynamic nature of psychotic disorders have been widely recognized [1]. The dimensional approach to the definition of psychotic spectrum disorders introduced in the *Diagnostic and Statistical Manual of Mental Disorders*, Fifth Edition (*DSM*-5) [2] is an attempt to account for the heterogeneity of psychopathology symptoms and signs in psychosis as well as their dynamic nature. Psychopathology dimensions are clusters of homogenous symptoms and signs that vary independently within every patient and can follow a unique dynamic pattern in time, which can in turn be used to design optimal treatment strategies. Most currently used classes of medications already target specific psychopathological dimensions (antipsychotic medications targeting positive and manic symptoms, antidepressants targeting depressive symptoms), while new medications are being tested, targeting other dimensions such as cognitive symptoms [3].

The influence of the dimensional approach is also apparent in the redefinition of well-established clinical symptom assessment tools, such as the Positive and Negative Syndrome Scale (PANSS) [4]. The original PANSS measured the positive and negative symptom scales and the general psychopathology scale, but recent work highlighted the effectiveness of a 5-factor solution [5]. In a meta-analysis of 45 studies using factor analysis of PANSS [6], the authors propose a consensus of the 5-factor solution for PANSS. The factors, following the pentagonal model for the dimensions of psychosis that has been adopted in *DSM*-5, include the positive factor (matching the positive dimension), the negative factor (matching the negative dimension), the cognitive/disorganization factor (matching the cognitive dimension), the depression/anxiety factor (matching the depression dimension), and the excitement/hostility factor (matching the mania dimension). The 5-factor model of PANSS can serve as a useful clinical tool to quantitatively assess the psychotic symptom dimensions and their dynamic changes over time.

While clinical definitions and taxonomies of psychotic disorders continue to be refined, sole reliance on phenomenology remains problematic [7]. The search for “biomarkers” related to psychotic disorder diagnosis is one of the most elusive research objectives in the field [8,9]. The term biomarker is defined as “a characteristic that is objectively measured and evaluated as an indicator of normal biological processes, pathogenic processes, or pharmacologic responses to a therapeutic intervention” [10]. Perhaps an even more ambitious goal would be to monitor longitudinal biomarker variation to detect or predict changes in psychopathology dimensions, thus establishing a link between biological processes and clinical observations. An important advantage of longitudinal designs in biomarker research, when compared to case-control studies, is that repeated measures in time can lead to characteristic findings at the individual level. This could allow for early, case-specific clinical interventions, ensuring optimal outcomes following the concept of personalized or precision medicine [11].

A promising advancement in personalized medicine is patient monitoring via digital phenotyping, which is defined as the moment-by-moment quantification of the individual-level human phenotype in situ using data from smartphones, smartwatches, and other personal digital devices [12]. These phenotypes include intermittent active data, requiring the participation of the individual (such as speech and video recording during a task or interview, question and answer surveys, etc) and continuous passive data that do not require participation of the individual such as physical activity (via accelerometer), heart rate (via photoplethysmography), spatial trajectories (via GPS), and light exposure (via camera) among others [13,14]. Passively recorded digital phenotypes allow for truly continuous monitoring over large periods of time and thus have been used in recent studies to detect and predict relapse onset. The core idea is that during long periods of remission, or relative health, markers such as physical activity or heart rate form strong, distinct patterns or characteristics for each individual. It is then postulated that during an acute episode of relapse, or even beforehand, abrupt, detectable pattern breaks may occur. Methodologically, features extracted from passively collected data are used to train machine learning models, such as anomaly detection models or neural networks, which then predict relapse as a binary outcome. Relapse could refer to a psychotic episode ([15-19], see [20] for a review), a mood episode [21-23], or a depressive episode ([24-26], see [27] for a review). While this approach has resulted in marked advances in digital phenotyping research, it does not come without shortcomings. First, relapse presents heterogeneously with respect to symptoms as well as behavior. For example, in [15], relapse was linked to a sharp decrease in time spent at the primary location (indicating increased mobility-roaming), while other studies have reported that more severe negative symptoms were linked to increased time at home and reduced mobility [28]. Given the different study designs, these results are not directly comparable, but we refer to them to underscore the real possibility that patients could exhibit either behavioral pattern. A wide array of symptom-behavior combinations is grouped under the single label of relapse, and this interferes with predictive accuracy. Moreover, the nontransparent model coefficients, the impossibility of isolating specific predictors for specific outcomes, and the risk of overfitting or lack of generalizability [29] diminish the potential of these results to be implemented in real-world clinical practice.

The main objective of this study was to combine ideas and methodological concepts from the emerging field of digital phenotyping with the dimensional approach for psychopathology while using an interpretable linear mixed effects (LME) model analysis approach. Instead of attempting to predict a binary outcome such as relapse, which is inherently diverse in its manifestations, we investigated correlations between digital phenotypes and each of the 5 dimensions of PANSS. We used data from the e-Prevention project [30], in which heart rate and mobility data were continuously recorded via smartwatches for up to 2 years. In a preliminary report [31], we used a subsample of the complete dataset to show that specific phenotypes correlate with the positive and negative symptom scale scores of PANSS, as defined originally in [4]. Here, we used the complete e-Prevention database as well as the 5D model of PANSS as described in [6]. The 5-factor PANSS was deemed more appropriate than the traditional PANSS because each dimension in the 5-factor model is more precisely defined compared to the traditional scale, which encompasses only 3 components, with one being quite heterogeneous (general psychopathology). We used a simple analysis method via LME models, where digital phenotype data were aggregated monthly and aligned temporally with monthly PANSS scores. Given the exploratory nature of our study and the lack of prior digital phenotyping literature involving PANSS measurements as outcomes, we valued robust main effects and interpretability highly, despite the loss of temporal resolution. We tested whether longitudinal changes in each symptom dimension would correspond to digital phenotype variation. We also investigated the relation of the digital phenotypes and psychopathology dimensions to demographic, clinical, and medication variables recorded in the same patient group to test for their confounding effects on the relation of digital phenotypes to psychopathology dimensions.

## Methods

### Participants

The data for this study were derived from the e-Prevention study database, which included 38 patients with psychotic spectrum disorders (schizophrenia, schizoaffective, and bipolar disorder). All patients were sequentially referred by their treating psychiatrists in remission, and a diagnostic evaluation was performed at the intake interview by 2 trained psychiatrists using *DSM*-5 criteria. At the intake interview, we recorded demographic data including age, gender, marital status, birthplace, occupation, educational level, and BMI. Patients were screened for a history of medical, neurological, psychiatric, and developmental disorders; family psychiatric history; birth complications; and history of substance abuse (smoking, alcohol, cannabis, and other psychotropic substances) (Table 1). Verbal IQ scores were derived from the administration of the Vocabulary Test of Wechsler Intelligence Scales [32] (Table 1).

**Table 1.** Demographic, clinical, and medication data and time of follow-up for patients in the e-Prevention database (N=38).

	Values
Demographics	
Age (years)	
Mean (SD)	30.5 (7.3)
Range	19‐45
Age category (years), n (%)	
18‐30	19 (50)
31‐45	19 (50)
Gender, n (%)	
Male	26 (68.4)
Female	12 (31.6)
Marital status, n (%)	
Single	32 (84.2)
Married	6 (15.8)
Birthplace, n (%)	
Urban	34 (89.5)
Rural	4 (10.5)
Occupation, n (%)	
Unemployed	17 (44.7)
Employed	16 (42.1)
Student	5 (13.2)
Education (years)	
Mean (SD)	13.4 (2.2)
Range	9‐18
Education category (years), n (%)	
≤12	24 (63.2)
>12	14 (36.8)
Verbal IQ	
Mean (SD)	97 (13.8)
Range	70‐120
Verbal IQ category, n (%)	
≥70 to ≤100	20 (52.6)
>100 to ≤120	18 (47.4)
BMI, n (%)	
Normal (<25)	13 (34.2)
Overweight (25-30)	17 (44.7)
Obese (>30)	8 (21.1)
Clinical data	
Diagnosis, n	
Schizophrenia	21
Schizoaffective	2
Bipolar	15
Diagnostic category, n (%)	
Schizophrenia	23 (55.3)
Affective	15 (39.5)
Disorder duration (years)	
Mean (SD)	7.3 (6.4)
Range	0‐23
Duration category, n (%)	
≤5 years	19 (50)
>5 years	19 (50)
Family history, n (%)	
Yes	20 (52.6)
No	18 (47.4)
Birth complications, n (%)	
Yes	11 (28.9)
No	27 (71.1)
Alcohol use, n (%)	
Yes	14 (36.8)
No	24 (63.2)
Smoking, n (%)	
Yes	26 (68.4)
No	12 (31.6)
Medication	
Antipsychotics, n (%)	
Yes	34 (89.5)
No	4 (10.5)
Chlorpromazine equivalents	
Mean (SD)	518.6 (481.4)
Range	0‐2000
Antidepressants, n (%)	
Yes	17 (44.7)
No	21 (55.3)
Fluoxetine equivalents	
Mean (SD)	8.1 (13.5)
Range	0‐60
Benzodiazepines, n (%)	
Yes	7 (18.4)
No	31 (81.6)
Diazepam equivalents	
Mean (SD)	0.9 (4.8)
Range	0‐40
Mood stabilizers, n (%)	
Yes	20 (52.6)
No	18 (47.4)
Time of follow-up	
Time (months)	
Mean (SD)	11.8 (7.2)
Range	1‐26

For each patient, we recorded the duration of the psychotic disorder before entering the study for each patient and medications including antipsychotics, antidepressants, mood stabilizers, and benzodiazepines (Table 1). Antipsychotic medication dosage was transformed into chlorpromazine equivalents [33]. Antidepressant medication dosage was transformed into fluoxetine equivalents [34]. Finally, benzodiazepine dosage was transformed into diazepam equivalents [35].

### Ethical Considerations

Each participant provided written informed consent for participation in the study and monitoring of clinical and digital phenotypes after being given a full description of the study design and objectives. Moreover, participants provided a separate consent form allowing the use of their personal data for the purposes of the e-Prevention research project in compliance with the General Data Protection Regulation (GDPR, EU Regulation 2016/679). The e-Prevention project study protocol and participant consent forms were reviewed and approved by the ethics committee of the University Mental Health, Neurosciences and Precision Medicine Research Institute “COSTAS STEFANIS,” (aprroval number: 778/14-5-2019). No compensation was provided to participants.

### Monthly Clinical Interviews

For the total duration of their participation in the e-Prevention, patients agreed to visit the Psychiatry Department of Eginitio University Hospital once every month for an evaluation during which psychopathology (PANSS) [4], BMI, and medication status (including equivalent transformed dosage of antipsychotics, antidepressants, and benzodiazepines) were assessed, among other clinical parameters [29]. The dimensional categorization of the PANSS scores [6] was used to derive the following 5D scores: (1) positive dimension, (2) negative dimension, (3) cognitive/disorganization dimension, (4) depression/anxiety dimension, and (5) excitement/hostility dimension.

### Digital Phenotypes

The e-Prevention database includes digital phenotypes collected passively from patients via a commercial smartwatch (Samsung Gear S3). Patients agreed to wear the smartwatch for the total duration of the study, continuously day and night, excluding charging time and activities that might lead to hardware failure (taking a shower or swimming). Details about system architecture, smartwatch feature extraction, and preprocessing are described in detail in our previous reports [30,31,36,37].

The accelerometer and gyroscope 3D signals were used to derive the short-time energy (*STE*) of their Euclidean norm over each 5-minute time window using the following formula:


$$STE=\sum_{j}^{n}x_{j}^{2}$$

where *x* is the norm of the respective accelerometer or gyroscope signal, *j* is the index of each sample under summation, and *n* is the total number of samples within the time window. The short-time energy can be thought of as the traditional measure of total signal energy (ie, the sum of the squared signal values), which can intuitively be seen as a quantitative measure of activity across the window which is used for summing. This is close to the interpretation of the activity counts of the widely used ActiGraph devices, without using thresholding for getting the counts and then summing. For each 5-minute interval, we derived a short-time energy value from the accelerometer, which measures accelerometer motor activity, and from the gyroscope, which measures gyroscope motor activity.

Heart measurements via photoplethysmography included beats per minute and intervals between successive pulses (interpulse intervals). Interpulse intervals were processed for removal of artifacts, and the count of the preprocessed interpulse intervals, named normal-to-normal intervals, for each 5-minute window was derived. This measure of normalized heart rate is considered a proxy of sympathovagal balance, or the ratio of sympathetic to parasympathetic activity [38]. The series of interpulse interval values (in milliseconds) was used to calculate the root mean square of their successive differences for each 5-minute interval. This measure of heart interpulse variation is considered a proxy of parasympathetic heart activity [39].

The sleep:wake ratio per day and the mean number of total steps per minute for each day as a measure of locomotive motor activity were also recorded. To improve the robustness of these measurements, we considered data only for days for which we have 18 or more hours of recorded data.

Our dataset included 740 patient months (minimum 1 month, maximum 26 months; mean: 11.8 months for each of the 38 patients; Table 1). We excluded months for which fewer than 720 five-minute measurements of wake data or fewer than 480 five-minute measurements of sleep data were collected. For each patient, we calculated the monthly mean and SD for each digital phenotype, separately for periods of wakefulness and sleep (except for sleep:wake ratio and locomotive motor activity). These data are summarized in Table S1 in Multimedia Appendix 1, alongside data from clinical interviews regarding psychopathology or medication. We derived the following digital phenotypes for each participant for every month: (1) mean and SD of accelerometer motor activity during wakefulness and during sleep, (2) mean and SD of gyroscope motor activity during wakefulness and during sleep, (3) mean and SD of normalized heart rate during wakefulness and during sleep, (4) mean and SD of heart interpulse variation during wakefulness and during sleep, (5) mean and SD of sleep:wake ratio, and (6) mean and SD of locomotive activity.

### Statistical Analysis

#### Analysis of the Relation of Digital Phenotypes to PANSS Dimensions

We used LME model analysis to estimate the relation of each one of the 20 digital phenotypes (independent predictor) with each one of the 5 PANSS dimension scores (dependent variable) using the SPSS 26 software (IBM). LME analysis is suitable for the current dataset, since it includes a different number of repetitions (months of follow-up) for each participant. The analysis is based on [40] and follows 3 steps.

In the first step, we perform an exploratory random intercept LME analysis, using the basic LME model in which each digital phenotype was entered as a fixed effect covariate predicting each PANSS dimension score (dependent variable), and participant was entered as a random intercept effect. This model is equivalent to a repeated measures analysis of variance assuming equal variance for repeated measures, for each participant. The maximum likelihood method was used to estimate the model. A total of 20 exploratory LME model analyses were performed for each PANSS dimension (one for each of 20 digital phenotypes), resulting in 100 analyses. Since these were exploratory analyses, we aimed to explore all possible significant effects at the nominal level of *P* (.05) without correction. To reduce the probability of false positive effects, in those analyses with a *P* value <.05, we further checked for the effect size of the predictor variable. We used the marginal pseudo-*R*^2^ value (Rm) provided in the coefficients of determination section of the SPSS results output to compute Cohen *f*^2^ with the formula: Cohen *f*^2^=Rm/(1 – Rm). We then retained those results with a *P* value <.05 and effect size *f*^2^≥0.01 showing that the significant effect also had a nonnegligible effect size.

The second step was performed only in those cases where the first-step analysis resulted in significant effects with a nonnegligible effect size of the predictor variable. In the first step of the LME model, all random errors associated with the repeated values of the dependent variable for each participant (PANSS dimension scores) were assumed to be independent (having zero covariance) and have constant variance. Since our data are repeated measures collected over time on each participant, the errors for the dependent variable (PANSS score) cannot be assumed to be completely independent of each other. In this case, one can assume that adjacent observations on the same participant will have errors with a higher correlation than observations that are farther apart, so we specified a first-order autoregressive [AR(1)] covariance structure [40]. The comparison of the model with the AR(1) covariance structure for random errors with the model without this structure was done by a specific statistical test computing the −2 log likelihood difference between the larger model including the AR(1) parameter and the reduced model without this parameter. Based on likelihood ratio theory, this difference represents a test statistic which follows a chi-square distribution with 1 degree of freedom because the reduced model has one fewer parameter [40]. If the *P* value for this test was lower than .05, this confirmed that the larger step 2 model had a significantly better fit compared to the simpler step 1 model. Note that when introducing an AR(1) covariance structure, the error structure of the model changes. Residual variance is no longer a scalar, but rather a full covariance matrix, and calculating a marginal pseudo-*R*^2^ would require collapsing this matrix into a single value, which is not directly comparable to the value defined for step 1 models.

The third step was performed again only in cases where the first-step analysis resulted in significant effects with a nonnegligible effect size of the predictor variable. In this model, we added a random slope effect for the independent predictor variable. This model assumes that the relation of the independent predictor (digital phenotype) and the dependent variable (PANSS dimension) in time is different for different participants (thus, the different random slopes). We further defined the covariance type in SPSS as “unstructured,” which allows for the estimation of the variances of the random effects (random intercept and random slope) as well as their covariance [40]. The comparison of the model including the random slope parameter to the model without this parameter requires a modification of the likelihood test as explained in the previous section [40]. In the case of a model with random intercepts and random slopes (two variances and a covariance associated with the random effects, assuming an unstructured covariance structure for the random effects), if one of the variances is dropped from the model, the difference in log-likelihood values between the two models follows a mixture of chi-square distributions, with 1 and 2 degrees of freedom, and equal weight 0.5, and the *P* value is computed as the sum of the half *P* values for the chi-square tests for 1 and 2 degrees of freedom (*P* value=½ *P* value with 1 degree of freedom + ½ *P* value with 2 degrees of freedom). If the final *P* value was lower than .05, this indicated that the larger step 3 model had a significantly better fit than the step 1 or step 2 model.

As an additional robustness check, we compared the LME models with nonlinear random forest and Gaussian process regression models trained on the same digital phenotype data, with the same applied exclusion criteria, using version 4.5.1 of the R software (see the Machine Learning Sensitivity Analyses section in Multimedia Appendix 1 for a detailed description of the additional analysis).

#### Analysis of the Effects of Demographic, Clinical, Medication, and Time Variables on Digital Phenotypes and Psychopathology Dimensions and Analysis of Interactions

Random intercept LME models to test the effect of a set of demographic, clinical, medication, and time variables (Table 1) as independent predictor factors on each of the 20 digital phenotypes (dependent variables) as well as the 5 psychopathology dimensions of PANSS (dependent variables). We did not use more complex models with repeated measures and random slope variance components of the independent factor since all independent demographic and clinical variables were categorical with no random variation. The 2 medication variables (chlorpromazine and fluoxetine equivalents) were continuous covariates, but again, these variables did not have enough within-subject variation, and the use of repeated measures and random slope effects resulted in ill-posed models that did not converge to the criterion. In the analysis, we used 5 demographic and 6 clinical factors summarized in Table 1, excluding mood stabilizers due to heterogeneity in treatment and benzodiazepines because their use was only sporadic. Finally, we used the time in months that each participant was followed as an independent continuous covariate. We report those results with a *P* value <.05 and effect size *f*^2^≥0.01.

To test for interaction effects, we used the set of final models with significant effects of digital phenotypes on PANSS dimensions that were described in the previous section and added the demographic, clinical, medication, and time variables that were significant in predicting both the digital phenotype and PANSS dimension as independent predictors. This was done to reduce the number of models testing for possible interactions to those that were meaningful, namely those for which we already had evidence that both the digital phenotype and the confounding variable had significant effects on the predicted PANSS dimension score.

## Results

### Effects of Digital Phenotypes on PANSS Dimensions

The results of the first step, exploratory analysis for the relation of digital phenotypes to the PANSS dimensions using random intercept LME models, are presented in Table S2 in Multimedia Appendix 1. Table 2 presents the results of the application of the LME model selection for selected models that survived step 1 analysis.

**Table 2.** Final LME^a^ model selection for the relation of digital phenotypes to PANSS^b^ dimensions.

Digital phenotype	Estimate (SE)	*t* test (*df*)^c^	*P* value	*f* ^2^	LME model comparison		Best-fitting model
					Chi-square (*df*)	*P* value	
Positive dimension							
Normalized heart rate–wake (mean)	0.017 (0.009)	1.9 (35.1)	.07	0.021^d^			
Step 2 vs step 1					33.4 (1)	<.001	Autocorrelation
Step 3 vs step 2					17.9 (1)	<.001	Random slope
Normalized heart rate–sleep (mean)	0.016 (0.009)	1.9 (33.7)	.06	0.05^d^			
Step 2 vs step 1					16.1 (1)	<.001	Autocorrelation^d^
Step 3 vs step 2					37.5 (1)	<.001	Random slope
Heart interpulse variation–sleep (mean)	–0.014 (0.04)	–3.3 (570.7)	<.001	0.021			
Step 2 vs step 1					0.4 (1)	.53	Random intercept
Step 3 vs step 1					5.1 (1)	.36	Random intercept
Negative dimension							
Accelerometer motor activity–wake (mean)	–0.375 (0.119)	–3.1 (22.1)	.005	0.042			
Step 2 vs step 1					0.1 (1)	.75	Random slope
Step 3 vs step 1					10.9 (1)	.003	Random slope
Accelerometer motor activity–wake (SD)	–0.291 (0.121)	–2.4 (20)	.03	0.019			
Step 2 vs step 1					1.9 (1)	.17	Random slope
Step 3 vs step 1					11.8 (1)	.002	Random slope
Gyroscope motor activity–wake (mean)	–0.00045 (0.00016)	–2.8 (22.9)	.01	0.016^d^			
Step 2 vs step 1					1.6 (1)	.2	Autocorrelation
Step 3 vs step 2					7.2 (1)	.02	Random slope^e^
Locomotive activity (mean)	–0.00023 (0.00009)	–2.4 (17.2)	.03	0.016^d^			
Step 2 vs step 1					3.7 (1)	.05	Autocorrelation
Step 3 vs step 2					23.0 (1)	<.001	Random slope^e^
Cognitive/disorganization dimension							
Normalized heart rate–wake (SD)	–0.029 (0.008)	–3.5 (574.5)	<.001	0.013			
Step 2 vs step 1					1.8 (1)	.18	Random intercept
Step 3 vs step 1					3.7 (1)	.11	Random intercept
Depression/anxiety dimension							
Accelerometer motor activity–sleep (mean)	1.49 (0.529)	2.8 (564.4)	.005	0.017			
Step 2 vs step 1					1.8 (1)	.18	Random intercept
Step 3 vs step 1					3.7 (1)	.11	Random intercept
Accelerometer motor activity–sleep (SD)	0.475 (0.187)	2.5 (551.6)	.01	0.015			
Step 2 vs step 1					0.7 (1)	.4	Random intercept
Step 3 vs step 1					2.4 (1)	.21	Random intercept
Gyroscope motor activity–sleep (mean)	0.003 (0.001)	3.2 (564.5)	.001	0.022			
Step 2 vs step 1					1.4 (1)	.24	Random intercept
Step 3 vs step 1					2.5 (1)	.2	Random intercept
Gyroscope motor activity–sleep (SD)	0.001 (0.00026)	2.8 (569.2)	.005	0.017			
Step 2 vs step 1					0.2 (1)	.65	Random intercept
Step 3 vs step 1					2.5 (1)	.2	Random intercept
Excitement/hostility dimension							
Accelerometer motor activity–sleep (SD)	0.548 (0.172)	3.2 (469.7)	.002	0.031			
Step 2 vs step 1					0.7 (1)	.4	Random intercept
Step 3 vs step 1					2.5 (1)	.2	Random intercept
Gyroscope motor activity–sleep (mean)	0.002 (0.001)	2.2 (497)	.03	0.013			
Step 2 vs step 1					2.7 (1)	.1	Random intercept
Step 3 vs step 1					4.7 (1)	.06	Random intercept
Gyroscope motor activity–sleep (SD)	0.001 (0.00024)	3.2 (507.7)	.001	0.029			
Step 2 vs step 1					1.6 (1)	.2	Random intercept
Step 3 vs step 1					1.1 (1)	.43	Random intercept
Normalized heart rate–wake (mean)	0.017 (0.009)	1.9 (20.28)	.07	0.026			
Step 2 vs step 1					1.7 (1)	.19	Random slope
Step 3 vs step 1					13.2 (1)	<.001	Random slope
Normalized heart rate–sleep (mean)	0.013 (0.004)	3.2 (368.2)	.001	0.044			
Step 2 vs step 1					1.1 (1)	.29	Random intercept
Step 3 vs step 1					4.7 (1)	.06	Random intercept
Sleep:wake ratio (mean)	–0.601 (0.222)	–2.7 (562)	.007	0.013			
Step 2 vs step 1					0.2 (1)	.16	Random intercept
Step 3 vs step 1					1.0 (1)	.6	Random intercept

a LME: linear mixed effects.

b PANSS: Positive and Negative Syndrome Scale.

c All *t* tests were 2-tailed.

d Approximation; refers to the effect size (*f*2) from the step 1 models.

e The random slope model did not converge, so we included the autocorrelation plus random slope, which resulted in model conversion.

Table 2 presents the results for the best-fitting LME model analysis selected after the 3-step process described in the methods section. Selected models of digital phenotypes predicting each PANSS dimension score are grouped for each PANSS dimension. In each model, we present the estimated coefficient for the fixed effect of the digital phenotype and its corresponding SE, as well as the *t* value and degrees of freedom (*df*), the *P* value, and the effect size (*f*^2^) for this fixed effect. In the last 3 columns, we present the *X*^2^ and corresponding *P* value for the comparison of the −2 log-likelihood of the different models at each step of the analysis (methods) and the best-fitting model.

In our LME models, increases in positive symptom dimension scores were related to increases in mean normalized heart rate during wakefulness and during sleep (Table 2). LME models including autocorrelation as well as random slope effects had significantly better fits than the simple random intercept-only models. Using these models, the relation of normalized heart rate during wakefulness and sleep to the PANSS positive dimension became nonsignificant (Table 3). Additionally, increasing positive symptom dimension scores were related to decreasing mean heart interpulse variation during sleep. More complex LME models (autocorrelation as well as random slope effects) did not lead to improved fit (Table 2), so the simpler model was retained.

**Table 3.** Significant effects of demographic and clinical variables on digital phenotypes.

	Mean (SE)	*t* test (*df*) or *F* test (*df*)^a^^,^^b^	*P* value	*f* ^2^
Gyroscope motor activity–wake (mean)		2.44 (36.3)	.02	0.14
Female	4354.6 (394.6)			
Male	3202.8 (259.9)			
Gyroscope motor activity–wake (SD)		2.32 (37)	.03	0.11
Schizophrenia	4066.3 (326.4)			
Bipolar	5260.6 (397.1)			
Gyroscope motor activity–sleep (SD)		2.43 (36.2)	.02	0.13
Female	1065.1 (119.1)			
Male	717.9 (78.5)			
Normalized heart rate–wake (mean)		3.18 (36)	.003	0.2
Smoking	369.5 (3.4)			
Nonsmoking	350.6 (4.8)			
Normalized heart rate–sleep (mean)		2.12 (36.5)	.04	0.11
Smoking	285.3 (9.1)			
Nonsmoking	308.9 (6.4)			
Heart interpulse variation–wake (mean)		2.47 (37.7)	.03	0.1
Married	305 (12.1)			
Single	275.4 (5.3)			
Heart interpulse variation–wake (mean)		2.49 (38.2)	.02	0.11
Rural birthplace	245.2 (14.7)			
Urban birthplace	284 (34.7)			
Heart interpulse variation–wake (mean)		*4.19 (36.2)*	.02	0.19
Unemployed	272.8 (7.1)			
Employed	294.1 (7)			
Student	257.4 (12.4)			
Heart interpulse variation–wake (mean)		*5 (33.1)*	.01	0.12
Unemployed	92.7 (1.6)			
Employed	87.3 (1.4)			
Student	95.7 (1.6)			
Heart interpulse variation–wake (SD)		2.7 (33.4)	.01	0.086
Education ≤12 years	92.7 (1.3)			
Education >12 years	87 (1.6)			
Heart interpulse variation–wake (SD)		*7.2 (602)*	<.001	0.027
Normal weight	89 (1.4)			
Overweight	90.9 (1.3)			
Obese	87.6 (1.5)			
Heart interpulse variation–sleep (SD)		2.6 (35.1)	.01	0.1
Female	69.8 (3.7)			
Male	81.3 (2.5)			
Heart interpulse variation–sleep (SD)		2.3 (34.8)	.03	0.075
Schizophrenia	82.7 (2.77)			
Bipolar	72.9 (3.25)			
Locomotive activity (SD)		2.29 (38.1)	.03	0.062
Married	1712.9 (371.7)			
Single	2638.3 (157.4)			
Sleep:wake ratio (mean)		3 (34.5)	.005	0.12
Female	0.729 (0.078)			
Male	1.01 (0.053)			
Sleep:wake ratio (mean)		2.67 (38.4)	.01	0.096
Married	0.639 (0.115)			
Single	0.974 (0.049)			
Sleep:wake ratio (mean)		2.09 (40.3)	.04	0.056
Rural birthplace	1.2 (0.147)			
Urban birthplace	0.89 (0.05)			
Sleep:wake ratio (mean)		3.08 (36.3)	.004	0.12
Duration ≤5 years	1.06 (0.062)			
Duration >5 years	0.786 (0.063)			
Sleep:wake ratio (SD)		2.5 (30.3)	.02	0.016
Female	0.32 (0.058)			
Male	0.501 (0.044)			
Sleep:wake ratio (SD)		2.6 (28.1)	.02	0.018
Married	0.226 (0.087)			
Single	0.474 (0.038)			

a Values in italics indicate *F* values.

b All *t* tests were 2-tailed.

Increases in negative symptom dimension scores were related to decreases in accelerometer motor activity (mean and SD), as well as decreases in mean locomotive activity and mean gyroscope motor activity during wakefulness. Table 3 presents the results of the application of the LME model selection. The more complex LME models had significantly better fits than the original random intercept models in all cases. Using these models, all the relations that were selected in the first-step analysis remained significant (Table 2).

Across repeated monthly assessments, higher cognitive/disorganization scores were associated with lower monthly variability (SD) of normalized heart rate during wakefulness. The more complex LME models did not provide a significantly better fit compared to the simpler step 1 model.

Increasing depression/anxiety symptom dimension scores were related to increasing mean accelerometer and gyroscope motor activity as well as their respective monthly variability (SD) during sleep (Table 3). The more complex step 2 and step 3 LME models were not significantly better compared to the step 1 model, as shown in Table 2.

Lastly, increasing excitement/hostility symptom dimension scores were associated with increasing mean gyroscope motor activity and increasing variability (SD) of accelerometer and gyroscope motor activity during sleep. There was also a relation between this symptom dimension and increasing mean normalized heart rate during wakefulness and during sleep, as well as a decreasing mean sleep:wake ratio (Table 2). The more complex step 2 and step 3 LME models were not significantly better compared to the step 1 models, except for the mean normalized heart rate during wakefulness. In that case, the more complex LME model (random slope) had a significantly better fit and was retained (Table 2). In this model, the effect of normalized heart rate during wakefulness on the excitement/hostility dimension score was not significant.

### Effects of Demographic, Clinical, Medication, and Time Variables

The results of the first-step LME model analysis for the effects of demographic, clinical, medication, and time variables on the digital phenotypes and on psychopathology dimensions are presented in Tables S3 and S4, respectively, in Multimedia Appendix 1, while significant results with an effect size >0.01 are further elaborated in Tables3  4.

**Table 4.** Significant effects of antipsychotic medication and time on digital phenotypes.

	Estimate (SE)	*t* test (*df*)^a^	*P* value	*f* ^2^
Antipsychotic medication				
Accelerometer motor activity–wake (SD)	−0.001 (0.00016)	3.62 (633)	<.001	0.023
Gyroscope motor activity–wake (SD)	−0.402 (0.151)	2.66 (628)	.008	0.014
Normalized heart rate–wake (mean)	0.005 (0.002)	2.99 (612)	.003	0.018
Heart interpulse variation–sleep (SD)	0.004 (0.002)	1.97 (.519)	.049	0.013
Locomotive activity (mean)	−0.61 (0.25)	2.41 (598)	.02	0.01
Sleep:wake ratio (mean)	0.00015 (0.00005)	3.5 (441.5)	<.001	0.041
Time of follow-up (months)				
Heart interpulse variation–wake (SD)	0.257 (0.046)	5.61 (605.5)	<.001	0.033

a All *t* tests were 2-tailed.

Table 3 presents the results of linear mixed model analyses for the significant effects of demographic and clinical factors on digital phenotypes that also had an effect size >0.01 (selected from Table S3 in Multimedia Appendix 1). The LME model estimated mean and SE for each value of the factor are presented in column 2, the *t* or *F* value (factors with 3 levels) for the fixed effect and its corresponding degrees of freedom (*df*) are presented in column 3, and finally, the *P* value for the fixed effect and its effect size are presented in columns 4 and 5.

Table 4 presents the results of the selected linear mixed model analyses of the significant effects of antipsychotic medication and time on digital phenotypes that also had an effect size >0.01 (selected from Table S2 in Multimedia Appendix 1). The LME model estimated mean and SE for the antipsychotic medication (chlorpromazine equivalents) and time (months) are presented in column 2, and the *t* value for the fixed effect and its corresponding degrees of freedom (*df*) are presented in column 3. The *P* value for the fixed effect is presented in column 4, and its effect size *f*^2^ in column 5.

Table 5 presents the results of linear mixed model analyses for the significant effects of demographic and clinical factors on psychopathology dimensions that also had an effect size >0.01 (selected from Table S3 in Multimedia Appendix 1). The LME model estimated mean and SE for each value of the factor are presented in column 2, the *t* or *F* value (factors with 3 levels) for the fixed effect and its corresponding degrees of freedom (*df*) are presented in column 3, and finally, the *P* value for the fixed effect is presented in column 4. All *t* tests were 2-tailed.

**Table 5.** Significant effects of demographic and clinical variables on psychopathology dimensions.

	Mean (SE)	*t* test (*df*) or *F* test (*df*)^a^^,^^b^	*P* value	*f* ^2^
Negative		2.33 (37.7)	.03	0.12
Female	13.4 (1.19)			
Male	16.8 (0.81)			
Negative		3.24 (39)	.002	0.19
Urban birthplace	15 (0.67)			
Rural birthplace	21.7 (2)			
Negative		*4.18 (37.7)*	.02	0.19
Employed	13.5 (1)			
Unemployed	17.2 (0.97)			
Student	17.7 (1.8)			
Negative		2.28 (36.01)	.03	0.12
Schizophrenia	17.3 (0.87)			
Bipolar	14.2 (1)			
Cognitive/disorganization		*5.06 (37.7)*	.01	0.2
Employed	9.2 (0.67)			
Unemployed	12.12 (0.65)			
Student	11.24 (1.2)			
Cognitive/disorganization		2.6 (36)	.01	0.16
Verbal IQ ≤100	11.82 (0.62)			
Verbal IQ >100	9.38 (0.7)			
Cognitive/disorganization		2.53 (36.1)	.02	0.14
Schizophrenia	11.59 (0.56)			
Bipolar	9.4 (0.66)			
Depression/anxiety		2.24 (35.9)	.03	0.082
Verbal IQ ≤100	6.5 (0.42)			
Verbal IQ >100	7.92 (0.48)			
Depression/anxiety		3.42 (36.1)	.002	0.18
Schizophrenia	6.44 (0.39)			
Bipolar	8.53 (0.47)			
Excitement/hostility		2.97 (36.1)	.005	0.12
Schizophrenia	6.1 (0.3)			
Bipolar	7.45 (0.38)			
Excitement/hostility		2.2 (38.2)	.03	0.061
Birth complications	7.46 (0.38)			
No birth complications	5.99 (0.32)			

a Values in italics indicate *F* values.

b All *t* tests were 2-tailed.

Table 6 presents the results of the selected linear mixed model analyses of the significant effects of antipsychotic medication and time on psychopathology dimensions (selected from Table S3 in Multimedia Appendix 1) with effect sizes >0.01. The LME model estimated mean and SE for the antipsychotic medication (chlorpromazine equivalents) and time (months) are presented in column 2, and the *t* value for the fixed effect and its corresponding degrees of freedom (*df*) are presented in column 3. The *P* value for the fixed effect is presented in column 4, and its effect size in column 5.

**Table 6.** Significant effects of medication and time on psychopathology dimensions.

	Estimate (SE)	*t* test (*df*)^a^	*P* value	*f* ^2^
Antipsychotic medication				
Positive	0.002 (0.0002)	9.06 (654.8)	<.001	0.19
Cognitive/disorganization	0.0008 (0.0002)	21.8 (669.8)	<.001	0.022
Antidepressant medication				
Excitement/hostility	−0.038 (0.01)	15.5 (465.4)	<.001	0.056
Time of follow-up (months)				
Positive	−0.06 (0.01)	−6.29 (650.3)	<.001	0.024

a All *t* tests were 2-tailed.

We then investigated potential confounding effects of demographic, clinical, medication, and time variables on the relations of digital phenotypes with PANSS dimensions (see Methods). To be eligible for inclusion in this analysis, a potential confounder had to be significant in predicting both the digital phenotype (Table S3 in Multimedia Appendix 1) and the PANSS dimension (Table S4 in Multimedia Appendix 1).

To begin with, the effect of mean normalized heart rate during wakefulness on the positive dimension was tested with antipsychotic medication as a confounding covariate. The interaction LME model analysis showed a nonsignificant interaction of mean normalized heart rate during wakefulness and antipsychotic medication on the PANSS positive dimension (*t*_95.6_=−1.86, *P*=.07) and separate significant effects of antipsychotic medication (*t*_98.3_=2.56, *P*=.01) and mean normalized heart rate during wakefulness (*t*_48_=2.32, *P*=.02). The effect of mean normalized heart rate during sleep on the PANSS positive dimension was tested with antipsychotic medication as a covariate. This LME model analysis showed a nonsignificant interaction of mean normalized heart rate during sleep and antipsychotic medication on the PANSS positive dimension (*t*_302.9_=1.08, *P*=.28) and nonsignificant effects of antipsychotic medication (*t*_297.8_=−.18, *P*=.85) and mean normalized heart rate during sleep (*t*_43.5_=1.2, *P*=.23). Finally, the effect of mean normalized heart rate during wakefulness on the positive dimension was tested with time of follow-up as a confounding covariate. The interaction LME model analysis showed a nonsignificant interaction of mean normalized heart rate during wakefulness and time on PANSS positive dimension (*t*_502.1_=−1.3, *P*=.2). The effect of time was not significant (*t*_505.4_=−0.94, *P*=.35), while the effect of mean normalized heart rate during wakefulness was significant (*t*_539.8_=2.71, *P*=.007).

The effect of the variability of accelerometer motor activity during wakefulness on the negative dimension of PANSS was tested with antipsychotic medication as a covariate. This LME model analysis showed a significant negative interaction of the variability of accelerometer motor activity during wakefulness and antipsychotic medication on PANSS negative dimension (*t*_231.6_=−3.15, *P*=.002) and a significant effect of antipsychotic medication on PANSS score (*t*_365.3_=3.98, *P*<.001) while the effect of the variability of accelerometer motor activity during wakefulness on PANSS negative dimension was not significant anymore (*t*_49.4_=−0.84, *P*=.4). The effect of mean gyroscope motor activity during wakefulness on the negative dimensions of PANSS was tested with gender as a cofactor. This LME model analysis showed a nonsignificant interaction of mean gyroscope motor activity during wakefulness and gender on PANSS negative dimension (*t*_31_=1.1, *P*=.28), while the effect of gender on PANSS score was significant (*t*_65.9_=2.85, *P*=.006), and the effect of mean gyroscope motor activity during wakefulness on PANSS negative dimension was also significant (*t*_34.1_=−2.95, *P*=.006). Finally, the effect of mean locomotive activity on the negative dimension of PANSS was tested with antipsychotic medication as a covariate. This LME model analysis showed a significant negative interaction of mean locomotive activity and antipsychotic medication on PANSS negative dimension (*t*_93.1_=−2.08, *P*=.04) and a significant effect of antipsychotic medication on PANSS negative dimension (*t*_271.2_=2.67, *P*=.008) while the effect of mean locomotive activity on PANSS negative dimension was not significant (*t*_34.8_=−0.88, *P*=.38). All *t* tests were 2-tailed.

## Discussion

### Digital Phenotypes and Psychotic Symptom Dimensions

In this study, we investigated the potential relation of continuously recorded digital phenotypes to the monthly variation of psychotic symptom dimensions measured with PANSS. We observed significant relations between psychotic symptomatology and digital phenotypes by applying the dimensional model composed of 5 symptom dimensions (positive, negative, cognitive/disorganization, depression/anxiety, and excitement/hostility). Notably, each symptom dimension was associated with a different set of digital markers, providing further evidence for the applicability of the dimensional model.

**Figure 1.**
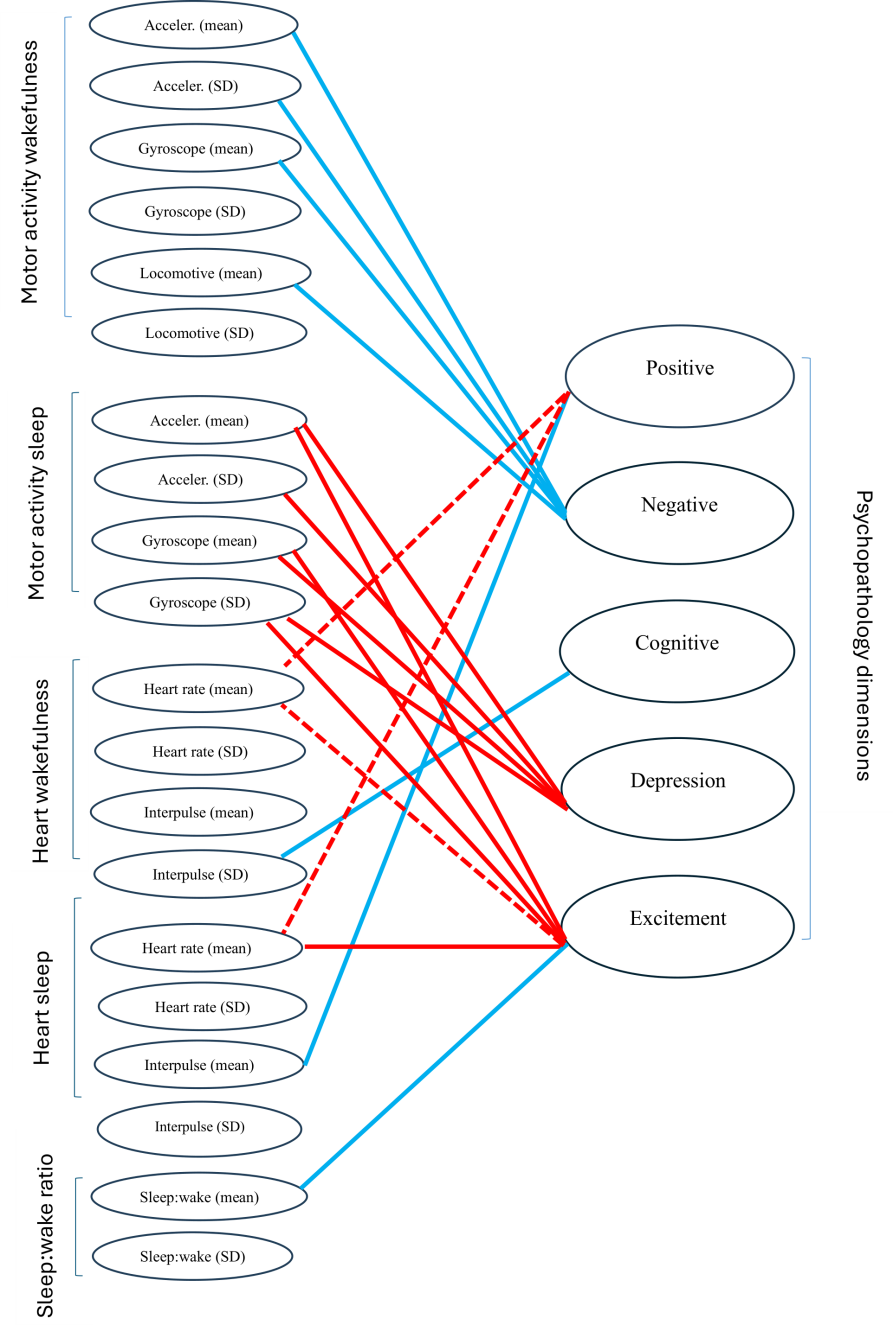
A line diagram showing significant relations between digital phenotypes (left) and psychopathology dimensions. Positive relations are presented with red color lines and negative relations with blue lines. Solid lines indicate significant relations, while dashed lines indicate marginally significant relations. Regarding the concept of marginally significant results, it refers to models that produced statistically significant results in the first step of the modeling procedure but failed to hold up in the next steps of the procedure, giving *P* values just over the .05 threshold (eg, .06). We mention these relationships as noteworthy, albeit nonsignificant. “Acceler.” is short for accelerometer, “heart rate” is short for normalized heart rate, and “interpulse” is short for interpulse variation.

Positive and excitement/hostility dimension symptoms were related to increased heart rate during wakefulness and sleep, indexing sympathovagal imbalance toward sympathetic activation, although only the increase of heart rate during sleep to increased excitement/hostility during sleep remained significant after application of more complex LME models including different slopes for this relation across participants. In our preliminary report [29], we showed that increased heart rate in wakefulness and sleep was related to the PANSS positive symptom scale score. Present results corroborate these initial findings in the sense that items from the PANSS original positive scale score are included in these 2 symptom dimensions (positive and excitement/hostility). A novel finding of this study was the specific relation of the positive symptom dimension to decreased heart interpulse variation during sleep, reflecting decreased parasympathetic activity, which was notably not shared with any other symptom dimension (Figure 1). The lack of universality of this relation across symptom dimensions is of particular interest given the vast body of literature ascertaining a connection between decreased vagal tone and schizophrenia and other psychotic disorders ([41-44], see [45,46] for a review). It is conceivable that decreased parasympathetic activation could be linked solely to the existence or exacerbation of positive symptomatology rather than the entire range of symptoms characterizing psychotic disorders, although this hypothesis requires further testing. From a neurobiological standpoint, this relation could be explained in association with the Neurovisceral Integration Model [47], in conjunction with the well-established dopamine hypothesis [48]. In short, cortical disinhibition (mostly in the prefrontal cortex) mediated by decreased vagal tone could subsequently lead to subcortical disinhibition, which could in turn result in dopaminergic hyperactivity and thus exacerbation of positive symptoms.

Increased negative dimension scores were related to decreased motility during wakefulness overall, marked by a decrease in accelerometer and gyroscope motor activity, a decrease in the variability of accelerometer motor activity, and a decrease in locomotive activity (Figure 1). A decrease in motility would be the expected overt behavior accompanying increased negative symptomatology [49,50]. It should be noted that in our preliminary report, we did not detect a relation of motility to the negative symptom scale score of PANSS. In that report, we observed that increased original PANSS negative scale scores were linked to decreased heart interpulse variation during wakefulness (indicating decreased parasympathetic activity) and increased sleep:wake ratio, findings which are in line with the literature cited above. While these effects were also detected in our study (Table S2 in Multimedia Appendix 1), they were excluded from further analysis due to their very small effect size (Cohen *f*^2^<0.01). This discrepancy between studies could be attributed to the different definitions of the original negative PANSS scale and the negative symptom dimension. Another plausible explanation could be that negative symptoms evolve much slower in time, and a longer period of observation would be needed to detect a robust relation to digital phenotypes measuring behavior.

Regarding the cognitive/disorganization symptom dimension, a significant, negative relation with the monthly variability of normalized heart rate during wakefulness was observed (Figure 1). This result could be indicative of disrupted sympathovagal balance related to cognitive/disorganization symptoms. In a related review [39], no studies examining heart-related autonomic dysregulation in mental processes such as attention or memory are cited. There are, however, 2 studies [51,52] in which social functioning is linked to a lower LF/HF ratio, indicative of an imbalance in autonomic regulation (higher sympathetic activity or lower parasympathetic activity). While results are not directly comparable due to differences in both the independent and dependent variables used, the alignment concerning autonomic dysregulation in this broader area of symptoms warrants further investigation.

The 2 mood-related dimensions (depression/anxiety and excitement/hostility) were related to increased gyroscope motor activity and its variability, as well as elevated accelerometer motor activity during sleep. The depression/anxiety dimension was also related to a higher variability of accelerometer motor activity during sleep. These findings are in line with established clinical observations, namely that increased motility is indicative of restless sleep, which in turn is characteristic of both depression and manic episodes [53-55]. Finally, the excitement/hostility dimension showed a unique negative relation to sleep:wake ratio, confirming that these symptoms are accompanied by less sleep (Figure 1).

### Interactions Among Digital Phenotypes, Clinical, Demographic, and Medication Factors, and Psychopathology Dimensions

The main objective of this study was to investigate the relations of digital phenotypes to psychopathology dimensions as well as demographic, clinical, medication, and time variables. Importantly, it could be hypothesized that these relations are confounded by demographic, clinical, medication, and time factors. If that were true, there should exist a direct connection among digital phenotypes and demographic, clinical, medication, and time variables, and among psychopathology dimensions and demographic, clinical, medication, and time variables simultaneously. While demographic variables were related to digital phenotypes (mainly gyroscope motor activity and heart interpulse variation), very few relations were detected between clinical variables and digital phenotypes. To conclude, the observed relations were generally not confounded by demographic or clinical factors. Finally, there were very few effects of time of follow-up on digital phenotypes.

Another finding with significant implications is that only 2 digital phenotypes varied significantly with diagnosis. The SD of gyroscope motor activity during wakefulness was larger for patients diagnosed with bipolar disorder compared to those with schizophrenia, and the monthly SD of heart interpulse variation during sleep was larger for patients with schizophrenia compared to those with bipolar disorder. If one compares these findings with the results concerning the relation of psychopathology dimensions to digital phenotypes, it is evident that digital phenotypes were more successful at dissociating the different psychopathology dimensions of psychotic disorders than the categorically defined syndromes of schizophrenia and bipolar disorder. These results further cement the dimensional model of psychosis [56].

As far as interaction effects among the 3 sets of variables (digital phenotypes; demographic, medication, and time factors; and psychopathology dimensions) are concerned, the only ones involving all sets of variables were those including antipsychotic medication or time confounders. Considering the significance of the link between normalized heart rate and the positive symptom dimension, we highlight that there was no significant interaction between normalized heart rate and either antipsychotic medication or time in predicting positive symptom dimension, conclusively ruling out antipsychotic medication and time as potential confounders. The same cannot be stated for the relation between decreased motility and negative symptoms. Significant interaction effects indicate that the observed reduction in motility is in part mediated by the effect of increased antipsychotic medication.

### Clinical Implications

From a clinical perspective, our results suggest that digital phenotyping could provide a complementary framework to traditional assessment via infrequent interviews, helping clinicians move toward dynamic, dimension-specific monitoring. Our approach can enrich existing, fully streamlined platforms such as mindLAMP [57], an application which collects active data (via surveys, questionnaires, etc) and passive data from a smartphone (GPS, accelerometer, call and text logs, etc). The application can be used for research purposes, while data can also be viewed by clinicians to plan interventions accordingly, assuming the patient provides consent. Smartwatches used in this study also offer bioinformation-rich heart rate data for real-time analyses, while requiring minimal effort from the patient, thus not hindering their everyday life and activities. Furthermore, current algorithms predict impending relapse with a significant but not reliable accuracy, leaving the possibility of false positives, which complicates clinical decision-making. The implementation of the dimensional approach also entails error; however, it offers flexibility to the clinician. By monitoring digital markers and obtaining predictions for specific symptom changes, a clinician can incorporate guidelines and experience on a case-by-case basis. For example, observing a monthly average change in heart rate of 60 would imply a change of 5 units in the PANSS positive dimension, potentially implicating persistent stress. The clinician could then adjust the treatment regimen, notify the patient with the intent of scheduling a visit, or continue to observe the digital marker trajectory. Although this effect size is small, further analyses incorporating more data could refine the models and produce far stronger symptom estimates. In summary, the differentiation of digital markers across psychopathology dimensions implies that clinicians could eventually tailor interventions based not only on observed symptomatology during infrequent visits, but also on objective, continuous data streams reflecting a patient’s everyday functioning.

Another finding with significant clinical implications is that symptom dimensions were more strongly associated with objective markers compared to static diagnostic labels. Given that symptom overlap is common among psychiatric disorders, it may be sensible to use longitudinal sensor-derived data as “digital biomarkers” to stratify patients not only by diagnosis but also by predominant symptom domains and their temporal dynamics. This could support more rational pharmacological decision-making, aid in evaluating treatment response in real-world settings, and provide an objective complement to subjective self-report scales during maintenance treatment. Over time, and with the accumulation of large-scale, real-world datasets, predictive models could be refined, enabling fully personalized clinical decisions, aligning with the ongoing efforts in precision medicine.

### Limitations

One potential limitation of this study is the small to medium sample size of patients. We believe that this limitation is countered by the long observation time, which allowed for a substantial amount of data collected for each patient. This resulted in a large database of digital phenotype and psychopathological estimation data (about 700 data points) that increased the power for detecting significant effects. That being said, the sample was relatively homogeneous, consisting of Greek outpatients aged 18‐45 years in the maintenance phase, which could compromise external validity. To address this, further studies are necessary across different regions and involving patients at various stages of the disorder.

Another limitation of this study lies in the implementation of a coarse-grained approach using LME modeling in our analysis. Although digital phenotypes were measured continuously in time, data from the entire month preceding each psychopathology measurement were collapsed into a single mean and variance value for each phenotype. While this approach serves to produce robust and interpretable results, which we valued highly in this study, it does have drawbacks. First, it is not sensitive to the short-term (ie, days or weeks) temporal evolution of digital phenotypes and thus cannot capture its potential significance in predicting psychopathology. Second, the LME approach cannot account for nonlinear effects and is not well suited to explore all the large number of total possible interaction effects. Third, LMEs can use specified covariance matrices [eg, AR(1)] but cannot fully capture complex autocorrelations which may be present in longitudinal datasets. Finally, monthly aggregation and LME application assume missingness at random, introducing bias in the case that missingness affects predictions. To address some of these concerns, we performed a basic sensitivity analysis, aiming to test our LME model approach against random forest and Gaussian Process Regression models, which can capture nonlinear relations. Both machine-learning approaches yielded modest explanatory power (*R*²<0.25 across PANSS dimensions) and similar patterns of predictor importance (Table S5 in Multimedia Appendix 1). These results suggest that potential nonlinear effects do not materially change the observed associations and support the interpretability-oriented LME modeling framework adopted here.

### Conclusions

This study adds to the mounting evidence in favor of the 5-factor model of PANSS, which can capture symptom profile heterogeneity, as well as symptom variation in time. Crucially, each psychopathology dimension was associated with a different set of digital markers, while those relations were not confounded by demographic or clinical variables. This finding constitutes a preliminary indication that biological changes such as decreased parasympathetic activity could underlie a narrower group of symptoms, rather than the entire psychotic disorder spectrum as assumed till now. Another important detail is that both behavioral markers and symptom severity vary in time, which seems inherently conflicting with static diagnostic labels and explains why only 2 of 19 used markers correlated with diagnosis. Lastly, we highlight the potential of smart devices (eg, watches or phones) and digital phenotypes not only as remote monitoring tools, but also as valuable research targets in the quest for objective markers in psychotic disorders.

## Supplementary material

10.2196/75774 Multimedia Appendix 1 Additional tables and machine learning sensitivity analyses*.*

## Abbreviations

AR(1): first-order autoregressive
*DSM-5*: *Diagnostic and Statistical Manual of Mental Disorders*, Fifth Edition
GDPR: General Data Protection Regulation
LME: linear mixed effects
PANSS: Positive and Negative Syndrome Scale

## References

[1] Cuesta MJ, Basterra V, Sanchez-Torres A, Peralta V (2009). Controversies surrounding the diagnosis of schizophrenia and other psychoses. Expert Rev Neurother.

[2] Biedermann F, Fleischhacker WW (2016). Psychotic disorders in DSM-5 and ICD-11. CNS Spectr.

[3] Javitt DC (2023). Cognitive impairment associated with schizophrenia: from pathophysiology to treatment. Annu Rev Pharmacol Toxicol.

[4] Kay SR, Fiszbein A, Opler LA (1987). The Positive and Negative Syndrome Scale (PANSS) for schizophrenia. Schizophr Bull.

[5] Wallwork RS, Fortgang R, Hashimoto R, Weinberger DR, Dickinson D (2012). Searching for a consensus five-factor model of the Positive and Negative Syndrome Scale for schizophrenia. Schizophr Res.

[6] Shafer A, Dazzi F (2019). Meta-analysis of the Positive and Negative Syndrome Scale (PANSS) factor structure. J Psychiatr Res.

[7] Pearlson GD, Clementz BA, Sweeney JA, Keshavan MS, Tamminga CA (2016). Does biology transcend the symptom-based boundaries of psychosis?. Psychiatr Clin North Am.

[8] Kraguljac NV, McDonald WM, Widge AS, Rodriguez CI, Tohen M, Nemeroff CB (2021). Neuroimaging biomarkers in schizophrenia. Am J Psychiatry.

[9] Wang B, Zartaloudi E, Linden JF, Bramon E (2022). Neurophysiology in psychosis: the quest for disease biomarkers. Transl Psychiatry.

[10] Puntmann VO (2009). How-to guide on biomarkers: biomarker definitions, validation and applications with examples from cardiovascular disease. Postgrad Med J.

[11] Quinlan EB, Banaschewski T, Barker GJ (2020). Identifying biological markers for improved precision medicine in psychiatry. Mol Psychiatry.

[12] Torous J, Kiang MV, Lorme J, Onnela JP (2016). New tools for new research in psychiatry: a scalable and customizable platform to empower data driven smartphone research. JMIR Ment Health.

[13] Cho CH, Lee T, Kim MG, In HP, Kim L, Lee HJ (2019). Mood prediction of patients with mood disorders by machine learning using passive digital phenotypes based on the circadian rhythm: prospective observational cohort study. J Med Internet Res.

[14] Onnela JP, Rauch SL (2016). Harnessing smartphone-based digital phenotyping to enhance behavioral and mental health. Neuropsychopharmacology.

[15] Ben-Zeev D, Brian R, Wang R (2017). CrossCheck: integrating self-report, behavioral sensing, and smartphone use to identify digital indicators of psychotic relapse. Psychiatr Rehabil J.

[16] Barnett I, Torous J, Staples P, Sandoval L, Keshavan M, Onnela JP (2018). Relapse prediction in schizophrenia through digital phenotyping: a pilot study. Neuropsychopharmacology.

[17] Adler DA, Ben-Zeev D, Tseng VWS (2020). Predicting early warning signs of psychotic relapse from passive sensing data: an approach using encoder-decoder neural networks. JMIR mHealth uHealth.

[18] Henson P, D’Mello R, Vaidyam A, Keshavan M, Torous J (2021). Anomaly detection to predict relapse risk in schizophrenia. Transl Psychiatry.

[19] Cohen A, Naslund JA, Chang S (2023). Relapse prediction in schizophrenia with smartphone digital phenotyping during COVID-19: a prospective, three-site, two-country, longitudinal study. Schizophrenia (Heidelb).

[20] Smyrnis A, Theleritis C, Ferentinos P, Smyrnis N (2024). Psychotic relapse prediction via biomarker monitoring: a systematic review. Front Psychiatry.

[21] Ebner-Priemer UW, Mühlbauer E, Neubauer AB (2020). Digital phenotyping: towards replicable findings with comprehensive assessments and integrative models in bipolar disorders. Int J Bipolar Disord.

[22] Faurholt-Jepsen M, Busk J, Vinberg M (2021). Daily mobility patterns in patients with bipolar disorder and healthy individuals. J Affect Disord.

[23] Tseng YC, Lin ECL, Wu CH, Huang HL, Chen PS (2022). Associations among smartphone app-based measurements of mood, sleep and activity in bipolar disorder. Psychiatry Res.

[24] Abbas A, Sauder C, Yadav V (2021). Remote digital measurement of facial and vocal markers of major depressive disorder severity and treatment response: a pilot study. Front Digit Health.

[25] Bai R, Xiao L, Guo Y (2021). Tracking and monitoring mood stability of patients with major depressive disorder by machine learning models using passive digital data: prospective naturalistic multicenter study. JMIR mHealth uHealth.

[26] Laiou P, Kaliukhovich DA, Folarin AA (2022). The association between home stay and symptom severity in major depressive disorder: preliminary findings from a multicenter observational study using geolocation data from smartphones. JMIR mHealth uHealth.

[27] Bufano P, Laurino M, Said S, Tognetti A, Menicucci D (2023). Digital phenotyping for monitoring mental disorders: systematic review. J Med Internet Res.

[28] Depp CA, Bashem J, Moore RC (2019). GPS mobility as a digital biomarker of negative symptoms in schizophrenia: a case control study. NPJ Digit Med.

[29] Rosen M, Betz LT, Schultze-Lutter F (2021). Towards clinical application of prediction models for transition to psychosis: a systematic review and external validation study in the PRONIA sample. Neurosci Biobehav Rev.

[30] Zlatintsi A, Filntisis PP, Garoufis C (2022). e-Prevention: advanced support system for monitoring and relapse prevention in patients with psychotic disorders analyzing long-term multimodal data from wearables and video captures. Sensors (Basel).

[31] Kalisperakis E, Karantinos T, Lazaridi M (2023). Smartwatch digital phenotypes predict positive and negative symptom variation in a longitudinal monitoring study of patients with psychotic disorders. Front Psychiatry.

[32] Russell AJ, Munro J, Jones PB, Hayward P, Hemsley DR, Murray RM (2000). The National Adult Reading Test as a measure of premorbid IQ in schizophrenia. Br J Clin Psychol.

[33] Gardner DM, Murphy AL, O’Donnell H, Centorrino F, Baldessarini RJ (2010). International consensus study of antipsychotic dosing. Am J Psychiatry.

[34] Hayasaka Y, Purgato M, Magni LR (2015). Dose equivalents of antidepressants: evidence-based recommendations from randomized controlled trials. J Affect Disord.

[35] Ashton CH (2002). Benzodiazepines: How They Work and How to Withdraw.

[36] Garoufis C, Zlatintsi A, Filntisis PP An unsupervised learning approach for detecting relapses from spontaneous speech in patients with psychosis.

[37] Efthymiou N, Retsinas G, Filntisis PP From digital phenotype identification to detection of psychotic relapses.

[38] Kim HG, Cheon EJ, Bai DS, Lee YH, Koo BH (2018). Stress and heart rate variability: a meta-analysis and review of the literature. Psychiatry Investig.

[39] Stogios N, Gdanski A, Gerretsen P (2021). Autonomic nervous system dysfunction in schizophrenia: impact on cognitive and metabolic health. NPJ Schizophr.

[40] West BT (2009). Analyzing longitudinal data with the linear mixed models procedure in SPSS. Eval Health Prof.

[41] Mujica-Parodi LR, Yeragani V, Malaspina D (2005). Nonlinear complexity and spectral analyses of heart rate variability in medicated and unmedicated patients with schizophrenia. Neuropsychobiology.

[42] Berger S, Boettger MK, Tancer M, Guinjoan SM, Yeragani VK, Bär KJ (2010). Reduced cardio-respiratory coupling indicates suppression of vagal activity in healthy relatives of patients with schizophrenia. Prog Neuropsychopharmacol Biol Psychiatry.

[43] Andersen EH, Lewis GF, Belger A (2018). Aberrant parasympathetic reactivity to acute psychosocial stress in male patients with schizophrenia spectrum disorders. Psychiatry Res.

[44] Huang WC, Liu WS, Chen TT, Chen WH, Huang WL (2020). Parasympathetic activity as a potential biomarker of negative symptoms in patients with schizophrenia. Asia Pac Psychiatry.

[45] Bär KJ (2015). Cardiac autonomic dysfunction in patients with schizophrenia and their healthy relatives – a small review. Front Neurol.

[46] Montaquila JM, Trachik BJ, Bedwell JS (2015). Heart rate variability and vagal tone in schizophrenia: a review. J Psychiatr Res.

[47] Thayer JF, Lane RD (2009). Claude Bernard and the heart-brain connection: further elaboration of a model of neurovisceral integration. Neurosci Biobehav Rev.

[48] Howes OD, Kapur S (2009). The dopamine hypothesis of schizophrenia: version III—the final common pathway. Schizophr Bull.

[49] Cella M, Okruszek Ł, Lawrence M, Zarlenga V, He Z, Wykes T (2018). Using wearable technology to detect the autonomic signature of illness severity in schizophrenia. Schizophr Res.

[50] Gorczynski P, Vancampfort D, Patel H (2018). Evaluating correlations between physical activity, psychological mediators of physical activity, and negative symptoms in individuals living with psychosis and diabetes. Psychiatr Rehabil J.

[51] Jáuregui OI, Costanzo EY, de Achával D (2011). Autonomic nervous system activation during social cognition tasks in patients with schizophrenia and their unaffected relatives. Cogn Behav Neurol.

[52] Kim Y, Kwon A, Min D, Kim S, Jin MJ, Lee SH (2019). Neurophysiological and psychological predictors of social functioning in patients with schizophrenia and bipolar disorder. Psychiatry Investig.

[53] Steardo L, de Filippis R, Carbone EA, Segura-Garcia C, Verkhratsky A, De Fazio P (2019). Sleep disturbance in bipolar disorder: neuroglia and circadian rhythms. Front Psychiatry.

[54] Hombali A, Seow E, Yuan Q (2019). Prevalence and correlates of sleep disorder symptoms in psychiatric disorders. Psychiatry Res.

[55] Basquin L, Maruani J, Leseur J (2024). Study of the different sleep disturbances during the prodromal phase of depression and mania in bipolar disorders. Bipolar Disord.

[56] Reininghaus U, Böhnke JR, Hosang G (2016). Evaluation of the validity and utility of a transdiagnostic psychosis dimension encompassing schizophrenia and bipolar disorder. Br J Psychiatry.

[57] mindLAMP. The Division of Digital Psychiatry at BIDMC.

