# Supplementary material for: Smartwatch-Derived Digital Phenotypes Relate to Psychopathology Dimensions in Patients With Psychotic Spectrum Disorders: Longitudinal Observational Study
Source: JMIR Ment Health. 2025 Dec 12;12:e75774. doi: 10.2196/75774 (PMC12700515; doi:10.2196/75774)
Supplement: Multimedia Appendix 1 [file mental-v12-e75774-s001.docx]

**Table S1.** Data base of PANSS and digital phenotype data used in the analysis.

|  | **N (% total)** | **Mean (SD)** | **Range** |
| --- | --- | --- | --- |
| **PANSS dimensions** | | | |
| Positive | 677(91.5%) | 6.7 (2.8) | 4-17 |
| Negative | 677 (91.5%) | 15.3 (4.9) | 7-29 |
| Cognitive/Disorganization | 677 (91.5%) | 10.4 (3.3) | 5-22 |
| Depression/Anxiety | 677 (91.5%) | 6.9 (2.4) | 3-14 |
| Excitement/Hostility | 677 (91.5%) | 6.4 (2.1) | 4-14 |
| **Accelerometer Motor Activity** | | | |
| Accelerometer Motor Activity-Wake (M) | 635 (85.8%) | 6.217 (2.447) | 1.628-16.902 |
| Accelerometer Motor Activity-Wake (SD) | 635 (85.8%) | 7.05 (2.278) | 2.564-16.001 |
| Accelerometer Motor Activity-Sleep (SD) | 629 (85%) | 0.509 (0.233) | 0.186-1.622 |
| Accelerometer Motor Activity-Sleep (SD) | 629 (85%) | 1.435 (0.699) | 0.551-4.548 |
| **Gyroscope Motor Activity** | | | |
| Gyroscope Motor Activity-Wake (M) | 635 (85.8%) | 3759.5 (1659.1) | 702-10534.5 |
| Gyroscope Motor Activity-Wake (SD) | 635 (85.8%) | 4728.9 (1970.8) | 1208.8-12440.8 |
| Gyroscope Motor Activity-Sleep (M) | 629 (85%) | 248.4 (143.4) | 73.9-913.4 |
| Gyroscope Motor Activity-Sleep (SD) | 629 (85%) | 815.5 (469.9) | 246.2-3017.5 |
| **Normalized Heart Rate** | | | |
| Normalized Heart Rate-Wake  (M) | 621 (83.9%) | 360.7 (21.04) | 270.3-444.3 |
| Normalized Heart Rate-Wake (SD) | 621 (83.9%) | 42.2 (12.3) | 20.8-113.9 |
| Normalized Heart Rate-Sleep  (M) | 622 (84%) | 299.4 (36.5) | 217.3-400.4 |
| Normalized Heart Rate-Sleep (SD) | 622 (84%) | 33.5 (10.5) | 14.8-137.3 |
| **Heart Inter-pulse Variation** | | | |
| Heart Inter-pulse Variation-Wake (M) | 621 (83.9%) | 209.4 (27.6) | 113.4-265.7 |
| Heart Inter-pulse Variation-Wake (SD) | 621 (83.9%) | 66.2 (7.7) | 47.4-93.5 |
| Heart Inter-pulse Variation-Sleep (M) | 622 (84%) | 87.7 (23.3) | 33.6-189.9 |
| Heart Inter-pulse Variation-Sleep (SD) | 622 (84%) | 56.3 (11.9) | 32.6-107.5 |
| **Locomotive Activity** | | | |
| Locomotive Activity (M) | 600 (81%) | 5008.1 (3363.9) | 328.5-25127.7 |
| Locomotive Activity (SD) | 600 (81%) | 2526.5 (1373.1) | 117.5-9005.7 |
| S**leep Wake Ratio** | | | |
| Sleep/Wake Ratio (M) | 600 (81%) | 0.89 (0.41) | .0001-3.41 |
| Sleep/Wake Ratio (SD) | 600 (81%) | 0.43 (0.686) | .000005-13.5 |

This table presents the database for the patient-month PANSS dimension score and digital phenotype data that were used in the current analysis. The number of retained month-patient data values for each PANSS dimension score or digital phenotype and percentage of total data (740) are shown in column 2, the mean and standard deviation (SD) in column 3 and the range in column 4.

**Table S2.** Random intercept LME model analysis of the relation of PANSS symptom dimensions to digital phenotypes.

| **Digital phenotype** | **Positive** | **Negative** | **Cognitive/**  **Disorganization** | **Depression/**  **Anxiety** | **Excitement/**  **Hostility** |
| --- | --- | --- | --- | --- | --- |
| Accelerometer Motor Activity-Wake (M) | .863 | **<.001(.018)** | .169 | .106 | .373 |
| Accelerometer Motor Activity-Wake (SD) | .547 | **<.001(.014)** | .166 | .201 | .191 |
| Accelerometer Motor Activity-Sleep (SD) | .717 | .037(.004) | .075 | **.005(.017)** | .098 |
| Accelerometer Motor Activity-Sleep (SD) | .772 | .913 | .201 | **.012(.015)** | **.002(.031)** |
| Gyroscope Motor Activity-Wake (M) | .940 | **.001(.016)** | .454 | .142 | .652 |
| Gyroscope Motor Activity-Wake (SD) | .590 | .014(.006) | .731 | .238 | .759 |
| Gyroscope Motor Activity-Sleep (M) | .902 | .913 | .085 | **.001(.022)** | **.030(.013)** |
| Gyroscope Motor Activity-Sleep (SD) | .714 | .070 | .316 | **.005(.017)** | **.001(.029)** |
| Normalized Heart Rate-Wake (M) | **<.001(.021)** | .453 | .109 | .342 | **.021(.013)** |
| Normalized Heart Rate-Wake (SD) | .100 | .661 | **<.001(.013)** | .500 | .593 |
| Normalized Heart Rate-Sleep (M) | **<.001(.05)** | .474 | .059 | .402 | **.001(.044)** |
| Normalized Heart Rate-Sleep (SD) | .329 | .728 | .008(.006) | .045(.008) | .088 |
| Heart Inter-pulse Variation-Wake (M) | .222 | .028(.007) | .386 | .092 | .522 |
| Heart Inter-pulse Variation-Wake (SD) | .641 | .947 | .161 | .051 | .518 |
| Heart Inter-pulse Variation-Sleep (M) | **<.001(.021)** | .407 | .8 | .155 | .122 |
| Heart Inter-pulse Variation-Sleep (SD) | .217 | .509 | .385 | .350 | .232 |
| Locomotive Activity (M) | .499 | **<.001(.016)** | .078 | .073 | .934 |
| Locomotive Activity (SD) | .463 | .018(.003) | .012(.003) | .632 | .609 |
| Sleep/Wake Ratio (M) | .208 | .002(.006) | .975 | .862 | **.007(.013)** |
| Sleep/Wake Ratio (SD) | .538 | .870 | .804 | .458 | .205 |

Table S2 presents the results of the first step analysis of the relation of digital phenotypes to PANSS dimension scores using the random intercept only linear mixed effects (LME) models. The p value of the fixed effect is shown for each analysis and Cohen f^2^ is shown in parentheses only in the cases where the p value was significant (<.05). The significant results with p <.05 and f^2^>=.01 that were selected for further analysis are marked in bold.

**Table S3.** LME model analysis of demographic clinical medication and time effects on digital phenotypes.

| **Accelerometer Motor Activity** | **Wake (M)** | **Wake (SD)** | **Sleep (M)** | **Sleep (SD)** |
| --- | --- | --- | --- | --- |
| Age category | .07 | .22 | .13 | .4 |
| Gender | .21 | .47 | .19 | .061 |
| Marital status | .25 | .86 | .64 | .34 |
| Birth Place | .21 | .57 | .37 | .39 |
| Occupation | .34 | .87 | .18 | .43 |
| Education category | .15 | .43 | .25 | .4 |
| Verbal IQ category | .5 | .19 | .1 | .23 |
| Body Mass Index | .62 | .44 | .006(.005) | .031(.004) |
| Diagnostic category | .77 | .1 | .23 | .15 |
| Duration category | .23 | .32 | .14 | .22 |
| Family History | .49 | .51 | .66 | .6 |
| Birth Complications | .39 | .97 | .99 | .72 |
| Alcohol use | .58 | .67 | .79 | .81 |
| Smoking | .92 | .77 | .57 | .46 |
| Chlorpromazine eq. | .37 | **<.001(.023)** | .13 | .97 |
| Fluoxetine eq. | .79 | .44 | .35 | .27 |
| Time (months) | .16 | .68 | <.001(.006) | .12 |
| **Gyroscope Motor Activity** | **Wake (M)** | **Wake (SD)** | **Sleep (M)** | **Sleep (SD)** |
| Age category | .1 | .34 | .13 | .4 |
| Gender | **.02(.14)** | .09 | .052 | **.02(.13)** |
| Marital status | .18 | .85 | .36 | .25 |
| Birth Place | .27 | .96 | .31 | .3 |
| Occupation | .37 | .94 | .33 | .65 |
| Education category | .17 | .38 | .37 | .54 |
| Verbal IQ category | .41 | .23 | .09 | .29 |
| Body Mass Index | .43 | .94 | .008(.005) | .035(.004) |
| Diagnostic category | .29 | **.02(.11)** | .17 | .16 |
| Duration category | .23 | .66 | .09 | .16 |
| Family History | .43 | .55 | .56 | .51 |
| Birth Complications | .24 | .51 | .9 | .78 |
| Alcohol use | .83 | .86 | .84 | .95 |
| Smoking | .92 | .35 | .69 | .69 |
| Chlorpromazine eq. | .31 | **.008(.014)** | **.**09 | .88 |
| Fluoxetine eq. | .69 | .94 | .15 | .2 |
| Time (months) | .13 | .37 | .003(.004) | .11 |
| **Normalized Heart Rate** | **Wake (M)** | **Wake (SD)** | **Sleep (M)** | **Sleep (SD)** |
| Age category | .88 | .6 | .56 | .43 |
| Gender | .31 | .28 | .38 | .67 |
| Marital status | .81 | .17 | .23 | .49 |
| Birth Place | .75 | .11 | .8 | .85 |
| Occupation | .92 | .61 | .71 | .61 |
| Education category | .17 | .57 | .56 | .51 |
| Verbal IQ category | .39 | .09 | .55 | .32 |
| Body Mass Index | .036(.008) | **.**011(.008) | .032(.005) | .12 |
| Diagnostic category | .1 | .34 | .36 | .48 |
| Duration category | .18 | .67 | .87 | .68 |
| Family History | .35 | .78 | .59 | .36 |
| Birth Complications | .81 | .25 | .96 | .23 |
| Alcohol use | .38 | .15 | .97 | .39 |
| Smoking | **.003( .2)** | .42 | **.041(.11)** | .48 |
| Chlorpromazine eq. | **.003(.018)** | .71 | .024(.006) | .28 |
| Fluoxetine eq. | .14 | .043(.008) | .1 | .95 |
| Time (months) | .009 (.004) | .26 | .97 | .45 |
| **Heart Inter-pulse Variation** | **Wake (M)** | **Wake (SD)** | **Sleep (M)** | **Sleep (SD)** |
| Age category | .64 | .29 | .96 | .97 |
| Gender | .59 | .63 | .34 | **.014(.1)** |
| Marital status | **.031(.1)** | .4 | .29 | .55 |
| Birth Place | **.017(.11)** | .052 | .24 | .51 |
| Occupation | **.023(.19)** | **.013(.12)** | .36 | .76 |
| Education category | .09 | **.01(.086)** | .42 | .57 |
| Verbal IQ category | .77 | .46 | .95 | .57 |
| Body Mass Index | .29 | **<.001( 0.027)** | .91 | .18 |
| Diagnostic category | .46 | .52 | .72 | **.028(.075)** |
| Duration category | .21 | .72 | .4 | .1 |
| Family History | .85 | .59 | .93 | .79 |
| Birth Complications | .45 | .57 | .13 | .08 |
| Alcohol use | .46 | .61 | .38 | .14 |
| Smoking | .09 | .1 | .25 | .83 |
| Chlorpromazine eq. | .38 | .21 | .54 | **.049(.013)** |
| Fluoxetine eq. | .39 | .14 | .18 | .08 |
| Time (months) | .79 | **<.001(.033)** | .67 | .68 |
| **Locmotive activity & sleep/wake ratio** | **Locomotive (M)** | **Locomotive (SD)** | **SWR(M)** | **SWR(SD)** |
| Age category | .2 | .72 | .092 | .53 |
| Gender | .62 | .72 | **.005(.12)** | **.019(.016)** |
| Marital status | .21 | **.027(.062)** | **.011(.096)** | **.015(.018)** |
| Birth Place | .59 | .97 | **.043(.056)** | .22 |
| Occupation | .69 | .7 | .38 | .5 |
| Education category | .36 | .38 | .07 | .17 |
| Verbal IQ category | .66 | .36 | .76 | .31 |
| Body Mass Index | .47 | .22 | .043(.008) | .07 |
| Diagnostic category | .47 | .52 | .12 | .91 |
| Duration category | .49 | .73 | **.004(.12)** | .25 |
| Family History | .91 | .79 | .25 | .99 |
| Birth Complications | .61 | .47 | .28 | .34 |
| Alcohol use | .48 | .92 | .74 | .88 |
| Smoking | .46 | .13 | .21 | .87 |
| Chlorpromazine eq. | **.016(.01)** | .11 | **<.001(.041)** | .38 |
| Fluoxetine eq. | .19 | .34 | .91 | .22 |
| Time (months) | .22 | .73 | .19 | .61 |

This table presents the results of the analysis of the relation of demographic/clinical/medication/time variables to digital phenotypes using the random intercept only linear mixed effects (LME) models. The p value of the fixed effect is shown for each analysis and Cohen f^2^ is shown in parentheses only in the cases where the p value was significant (<.05). The significant results with p <.05 and f^2^ >=.01 are marked in bold.

**Table S4.** LME model analysis of demographic, clinical, medication and time effects on PANSS dimensions.

|  | **Positive** | **Negative** | **Cognitive/ disorganization** | **Depression/ anxiety** | **Excitement/ hostility** |
| --- | --- | --- | --- | --- | --- |
| Age category | .77 | .3 | .47 | .68 | .92 |
| Gender | .23 | .**025(.12)** | .11 | .31 | .71 |
| Marital status | .84 | .14 | .27 | .72 | .8 |
| Birthplace | .07 | **.002(.19)** | .06 | .89 | .98 |
| Occupation cat. | .36 | **.023(.19)** | **.011(.2)** | .7 | .29 |
| Education cat. | .12 | .085 | .14 | .76 | .38 |
| Verbal IQ cat. | .37 | .47 | .**013(.16)** | .**031(.082)** | .88 |
| BMI | .002(.009) | .001(.007) | .31 | .56 | .18 |
| Diagnostic cat. | .12 | **.028(.12)** | .**016(.14)** | **.002(.18)** | **.005(.12)** |
| Duration category | .14 | .82 | .29 | .21 | .25 |
| Family History | .25 | .44 | .25 | .77 | .88 |
| Birth Complic. | .08 | .35 | .14 | .6 | .**034(.061)** |
| Alcohol use | .94 | .16 | .19 | .17 | .39 |
| Smoking | .76 | .74 | .15 | .52 | .12 |
| Chloropr/zine eq. | **<.001(.19)** | .021(.006) | **<.001(.022)** | .17 | .48 |
| Fluoxetine eq. | .87 | .16 | .94 | .7 | **<.001(.056)** |
| Time (months) | **<.001(.024)** | .21 | .006(.002) | .19 | .31 |

This table presents the results of the analysis of the relation of demographic/clinical/medication/time variables to PANSS dimension scores using the random intercept only linear mixed effects (LME) models. The p value of the fixed effect is shown for each analysis and Cohen f^2^ is shown in parentheses only in the cases where the p value was significant (<.05). The significant results with p <.05 and f^2^ >=.01 are marked in bold.

**Methods: Machine Learning Sensitivity Analyses**

**Overview**

To assess the robustness of the linear mixed-effects (LME) findings and check for potential nonlinear effects, we performed an additional sensitivity analysis using two non-linear regression tools: Random Forests (RF), an ensemble tree-based method that captures complex feature interactions, and Gaussian Process Regression (GPR), a non-parametric Bayesian approach that can flexibly model smooth nonlinear functions. The analysis used the same monthly-aggregated data set as the LME models (N = 740 patient-months across 38 participants) and the same exclusion criteria were applied. The same digital phenotypes were used as predictors for each model (wake and sleep separately), while the analysis was run with each of the five PANSS dimensions (positive, negative, cognitive/disorganization, depression/anxiety, and excitement/hostility) as outcomes. This procedure resulted in a total of 5 outcomes (dimensions) x 2 states (wake/sleep) x 2 modeling methods (RF and GPR) = 20 models (results presented in Table S5).

**Model Specification**

All analyses were conducted in R (version 4.5.1) using the packages *ranger*, *kernlab*, and *caret* in the *tidyverse* environment, for model fitting and validation. Two nonlinear regression approaches were implemented:

*Random Forest Regression (RF):*RF models were fitted using the *ranger* package with 500 trees and sampling without replacement. The number of variables randomly selected at each split was optimized using grid search within cross-validation. The RF approach was selected for its ability to model nonlinear relationships and higher-order interactions.

*Gaussian Process Regression (GPR):*
GPR models were implemented using the *kernlab* package with a radial basis function (RBF) kernel (i.e. Gaussian kernel). Kernel hyperparameters, including the characteristic length scale, variance, and noise term, were optimized via marginal likelihood maximization. This approach allows for flexible modeling of smooth nonlinear functions and provides explicit uncertainty estimates for predictions.

**Cross-Validation and Performance Metrics**

Model performance was evaluated using 10-fold cross-validation stratified by subject, to minimize data leakage from repeated measures. For each fold, predicted and observed PANSS dimension scores were compared using the R² (coefficient of determination), the RMSE (root mean squared error), and the MAE (mean absolute error). Values were averaged across folds (means and standard deviations are reported in Table S5). For RF models, we also reported the top three most important predictors explicitly.

**Results: Machine Learning Sensitivity Analyses**

*Model Performance*

Both nonlinear modeling approaches (RF and GPR) were evaluated across all five PANSS dimensions and both wake and sleep states. Overall, the models demonstrated low to moderate predictive performance, with generally low R² values (Table S5).

Among RF models, the highest explanatory power was observed for the negative dimension during wakefulness (R² = 0.20 ± 0.17) and for the positive dimension during sleep (R² = 0.13 ± 0.12). GPR models achieved similar or slightly higher fit in a few cases (e.g., negative dimension during wakefulness, R² = 0.24 ± 0.19), but differences were small and well within cross-validation variability.

Importantly, no dimension exhibited a consistent performance gain under GPR relative to RF or to the original LME analyses, suggesting that the relationships between digital phenotypes and symptom dimensions are predominantly linear in this dataset.

*Feature Importance*

Regarding feature importance, there was a strong convergence between modeling approaches (RF and LME). In the Positive dimension Heart Inter-Pulse Variability during sleep emerged as a top predictor in both RF and LME analyses. In the Negative dimension, reduced Accelerometer and Gyroscope Motor Activity during wakefulness were common to both frameworks. In the Depression/Anxiety and Excitement/Hostility dimensions, increased Accelerometer and Gyroscope activity during sleep were shared findings across LME and RF models, corresponding to restless or fragmented sleep patterns in mood-related symptomatology.

Overall, four of the five PANSS dimensions showed partial or complete overlap between LME-significant predictors and RF-ranked top features. The exception was the Cognitive/Disorganization dimension, where a different set of important predictors was identified by the RF model.

**Table S5.** Results from the sensitivity analysis, using Random Forest (RF) and Gaussian Process Regression (GPR) models.

| **PANSS dimension** | **State** | **Model** | **R² (mean ± SD)** | **RMSE (mean ± SD)** | **MAE (mean ± SD)** | **Top 3 predictors (M = mean*, standard deviation = SD)** |
| --- | --- | --- | --- | --- | --- | --- |
| Negative | Wake | RF | 0.201 ± 0.173 | 4.248 ± 0.589 | 3.447 ± 0.537 | Normalized Heart Rate M, Accelerometer M, Gyroscope M |
| Negative | Sleep | RF | 0.102 ± 0.139 | 4.981 ± 1.067 | 4.068 ± 0.866 | Heart Inter-Pulse Variability M, Accelerometer M, Normalized Heart Rate M |
| Negative | Wake | GPR | 0.240 ± 0.186 | 4.063 ± 0.507 | 3.353 ± 0.415 | n/a |
| Negative | Sleep | GPR | 0.058 ± 0.052 | 5.229 ± 1.079 | 4.363 ± 0.937 | n/a |
| Positive | Wake | RF | 0.020 ± 0.026 | 3.028 ± 0.731 | 2.388 ± 0.513 | Heart Inter-Pulse Variability M, Locomotive M, Gyroscope SD |
| Positive | Sleep | RF | 0.131 ± 0.117 | 3.059 ± 0.498 | 2.453 ± 0.313 | Heart Inter-Pulse Variability M, Gyroscope M, Normalized Heart Rate M |
| Positive | Wake | GPR | 0.030 ± 0.030 | 3.102 ± 0.747 | 2.425 ± 0.505 | n/a |
| Positive | Sleep | GPR | 0.098 ± 0.111 | 3.184 ± 0.602 | 2.524 ± 0.407 | n/a |
| Cognitive/ Disorganization | Wake | RF | 0.007 ± 0.005 | 3.348 ± 0.666 | 2.683 ± 0.630 | Locomotive M, Accelerometer M, Gyroscope SD |
| Cognitive/ Disorganization | Sleep | RF | 0.044 ± 0.050 | 3.559 ± 0.705 | 2.823 ± 0.516 | Gyroscope M, Heart Inter-Pulse Variability M, Normalized Heart Rate M |
| Cognitive/ Disorganization | Wake | GPR | 0.003 ± 0.004 | 3.532 ± 0.660 | 2.823 ± 0.566 | n/a |
| Cognitive/ Disorganization | Sleep | GPR | 0.098 ± 0.119 | 3.802 ± 0.785 | 3.051 ± 0.634 | n/a |
| Depression/ Anxiety | Wake | RF | 0.040 ± 0.050 | 2.703 ± 0.663 | 2.225 ± 0.588 | Accelerometer SD, Accelerometer M, Normalized Heart Rate SD |
| Depression/ Anxiety | Sleep | RF | 0.032 ± 0.064 | 2.711 ± 0.436 | 2.310 ± 0.408 | Accelerometer SD, Accelerometer M, Normalized Heart Rate SD |
| Depression/ Anxiety | Wake | GPR | 0.009 ± 0.005 | 2.718 ± 0.592 | 2.258 ± 0.550 | n/a |
| Depression/ Anxiety | Sleep | GPR | 0.042 ± 0.050 | 2.795 ± 0.400 | 2.372 ± 0.357 | n/a |
| Excitement/ Hostility | Wake | RF | 0.030 ± 0.032 | 2.215 ± 0.121 | 1.775 ± 0.119 | Accelerometer SD, Gyroscope M, Gyroscope SD |
| Excitement/ Hostility | Sleep | RF | 0.011 ± 0.005 | 2.348 ± 0.205 | 1.896 ± 0.223 | Accelerometer SD, Normalized Heart Rate M, Gyroscope SD |
| Excitement/ Hostility | Wake | GPR | 0.011 ± 0.015 | 2.204 ± 0.121 | 1.766 ± 0.044 | n/a |
| Excitement/ Hostility | Sleep | GPR | 0.015 ± 0.009 | 2.323 ± 0.210 | 1.879 ± 0.188 | n/a |

*Here, M and SD represent feature definitions, e.g. monthly Accelerometer M.
